# Sodium Alginate Modified Platinum Nanozymes With Highly Efficient and Robust Oxidase-Like Activity for Antioxidant Capacity and Analysis of Proanthocyanidins

**DOI:** 10.3389/fchem.2020.00654

**Published:** 2020-08-05

**Authors:** Shao-Bin He, Liu Yang, Xiu-Ling Lin, Hua-Ping Peng, Zhen Lin, Hao-Hua Deng, Wei Chen, Guo-Lin Hong

**Affiliations:** ^1^Fujian Key Laboratory of Drug Target Discovery and Structural and Functional Research, School of Pharmacy, Fujian Medical University, Fuzhou, China; ^2^Department of Laboratory Medicine, The First Affiliated Hospital of Xiamen University, Xiamen, China

**Keywords:** sodium alginate, platinum nanozyme, oxidase-like activity, antioxidant capacity, colorimetric, proanthocyanidins

## Abstract

Platinum nanozymes exhibiting highly efficient and robust oxidase-like activity are successfully synthesized and modified using sodium alginate (SA-PtNPs). According to a steady-state dynamic assay, Michaelis-Menton constant (*K*_*m*_) is calculated as 11.6 μM, indicating that the affinity of SA-PtNPs toward the substrate, 3, 3′, 5, 5′-tetramethylbenzidine (TMB), is high. It shows in the paper that SA-PtNPs exhibit a significant oxidant effect on substrate-O_2_ to produce ${\text{O}}_{2}^{•-}$ as an oxidase mimic. Moreover, the oxidase-like activity fluctuated slightly under changes in environmental pH and incubation time, implying that SA can increase the dispersibility and stability of PtNPs. A colorimetric assay for oligomeric proanthocyanidins (OPC) was realized given how few explorations of the former there are. We found that the significant inhibitory effect of OPC on the oxidase-like activity is due to the competitive effect between OPC and TMB for binding to the active site of SA-PtNPs, resulting in a color change. Under optimal conditions, the logarithmic value of the chromogenic difference (ΔA_450nm_) to OPC concentration was linear (4–32.5 μM, *r* = 0.999) with a limit of detection (LOD) of 2.0 μM. The antioxidant capacity of OPC obtained by the Soxhlet extraction method from grape seeds was 2.85 U/mg. The recovery from the experiment in which OPC was added to grape seeds ranged from 97.0 to 98.6% (RSDs of 0.5–3.4%), suggesting a high accuracy in OPC detection. These findings are important because OPC is an internationally recognized antioxidant that eliminates free radicals in the human body and, therefore, may prevent a variety of diseases. Thus, we envisage that this Pt nanozyme-based assay may be prevalent for antioxidant capacity evaluation and analytical applications.

## Introduction

Proanthocyanidin (PC) is a general term for a large class of polyphenolic compounds widely found in plants, red wine, as well as grape seeds (Da Silva et al., 1991; Fracassetti et al., 2013). Its oligomer, oligomeric proanthocyanidin (OPC), is an internationally recognized antioxidant that eliminates free radicals in humans (Weber et al., 2007). Therefore, OPC may provide disease prevention caused by free radicals such as cardiopathy, malignant tumor, and inflammation (Vaid et al., 2012; Coleman and Shaw, 2017; Chen et al., 2019). Thus, OPC is expected to extend its applications in clinical medicine and food health practices. However, owing to the complexity of OPC, there have not been enough studies done on OPC contents and antioxidant capacity. At present, most methods use high-performance liquid chromatography or mass spectrometry technology to analyze PC or OPC and are time-consuming, expensive, and technically demanding (Ortega et al., 2010; Dooren et al., 2018). To this end, it is of fundamental significance to establish a colorimetric method to evaluate antioxidant capacity and analyze PC or OPC; it should have superior visibility, be a time-saver, and be easy to operate.

Since 2007, when Yan's group first discovered that Fe_3_O_4_ magnetic nanoparticles have catalytic activity like horseradish peroxidase (Gao et al., 2007), researches of nanozymes (nanomaterials with enzyme-like activity) have been continuously deepened, and a series of nanozymes-based analytical assays have subsequently been obtained (Wei and Wang, 2013; Huang et al., 2019). Among the nanomaterials, platinum nanoparticles (PtNPs) have been explored to mimic several kinds of enzyme activities such as peroxidase, catalase, uricase activities, and so on (Dong et al., 2011; Fu et al., 2014; Li et al., 2015; Liu et al., 2015; He et al., 2019a). Meanwhile, the oxidase-like activity of PtNPs has elicited growing attention in recent years (Yu et al., 2014; Deng et al., 2017; He et al., 2019b). Unlike peroxidase, oxidase can catalyze a certain substrate in the absence of H_2_O_2_, having far-reaching implications for the study of oxidase mimics. Additionally, H_2_O_2_ is easily decomposed and loses its oxidizing power; this is also disadvantageous for its practical application. Inspired by the above, a new nanozyme with oxidase-like activity is far more propitious to design analytical assays with user-friendly control, stability, and dependability.

During the synthesis of nanomaterials, the role of ligand is also crucial. The protective effect of ligands enables people to successfully obtain a variety of nanomaterials. Moreover, the use of natural products as ligands instead of chemicals to stabilize nanoparticles can greatly improve biocompatibility and broaden their biomedical applications. To this end, efforts have been made to adjust the size and morphology of PtNPs to mimic enzyme-like activity by applying natural products (Deng et al., 2017, 2019; You et al., 2017; He et al., 2019a,b). Sodium alginate (SA) is a natural polysaccharide derived with a certain solubility, stability, and viscosity to form a gel, making them suitable for broad applications (Li et al., 2005; Mokhtari et al., 2017). Given the advantages of SA, the use of SA as a protective agent to modify nanomaterials not only enhances the dispersibility and stability but also provides the possibility of surface modification, nano-gel preparation, and potential applications in many fields (Travan et al., 2009).

**Scheme 1:**
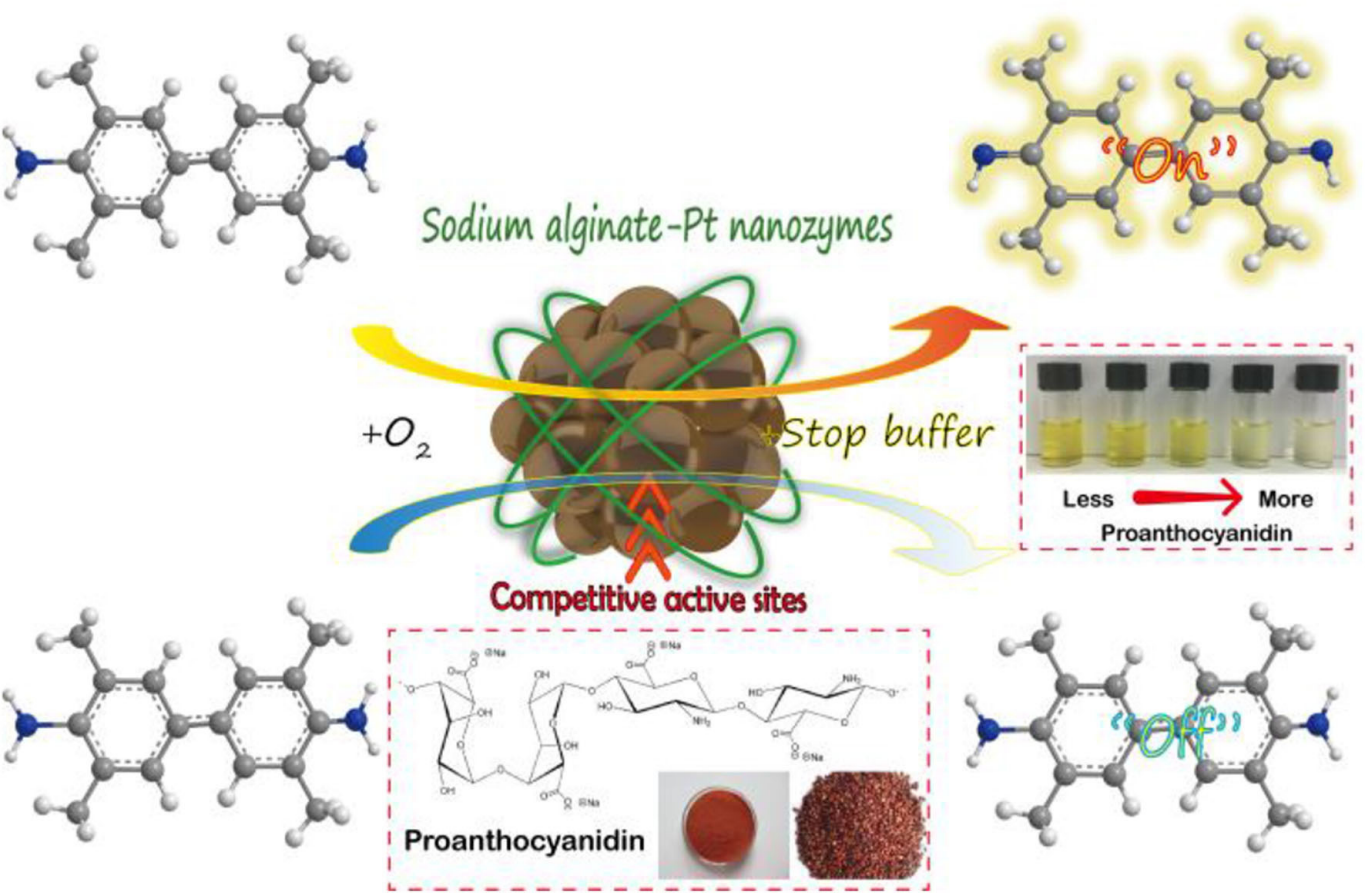
Sodium alginate modified platinum nanozymes with oxidase-like activity for antioxidant capacity and analysis of proanthocyanidins.

In this study, SA-PtNPs were prepared by a one-step reduction method using the biocompatible and non-toxic SA as a protective agent. SA-PtNPs have small particle size, good dispersion, and stability (5.9 ± 0.6 nm). Furthermore, SA-PtNPs have intrinsic oxidase activity that can rapidly catalyze the oxidation process of 3, 3′, 5, 5′-tetramethylbenzidine (TMB) leading to a color change. More significantly, the introduction of OPC in SA-PtNPs-catalyzed TMB system results in a competitive inhibition and colorless reaction. Thus, a colorimetric assay for OPC was established based on the excellent oxidase-like activity of SA-PtNPs and the antioxidant capacity of OPC (Scheme 1). This study presents a new preparation method for Pt nanozymes and shows a good prospect of application based on SA-PtNPs. On the other hand, this assay also provides a new insight for antioxidant capacity evaluation and opens a new avenue for OPC analysis.

## Experimental Part

### Synthesis of SA-PtNPs

The synthesis steps are as follows: SA (0.1 g) was added into 50 mL of 1% (v/v) acetic acid solution and completely stirred for about 15 min. Then, 2 mL H_2_PtCl_6_ (10 mM) was mixed with 47 mL SA solution. The mixture was vortex at ambient temperature for 30 min. One milliliters of newly prepared NaBH_4_ solution (70 mM) was added to the mixture and was completed within 5 min. The SA-PtNPs (78.03 mg/L) were obtained by stirring in the dark for 90 min. The SA-PtNPs was transferred in a dialysis bag and dialysed against water. The dialysis water was replaced after 1 h followed by frequent changes for 48 h. Finally, the SA-PtNPs were preserved constantly in darkness at 4°C.

### OPC Detection

Fifty μL of various concentrations of OPC, 50 μL of TMB (3 mM), and 30 μL of SA-PtNPs (15.61 mg/L) were added into 870 μL of phosphate buffer (pH = 4.5, 50 mM). The mixture was then incubated for 5 min at 37°C. Subsequently, 200 μL of H_2_SO_4_ (2 M) was introduced to the reaction as a stop buffer. Finally, the solution was transferred to a quartz cell for measurement at 450 nm. Other experimental part can be seen in Supplementary Material.

## Results and Discussion

### Characterization of SA-PtNPs

**Figure 1:**
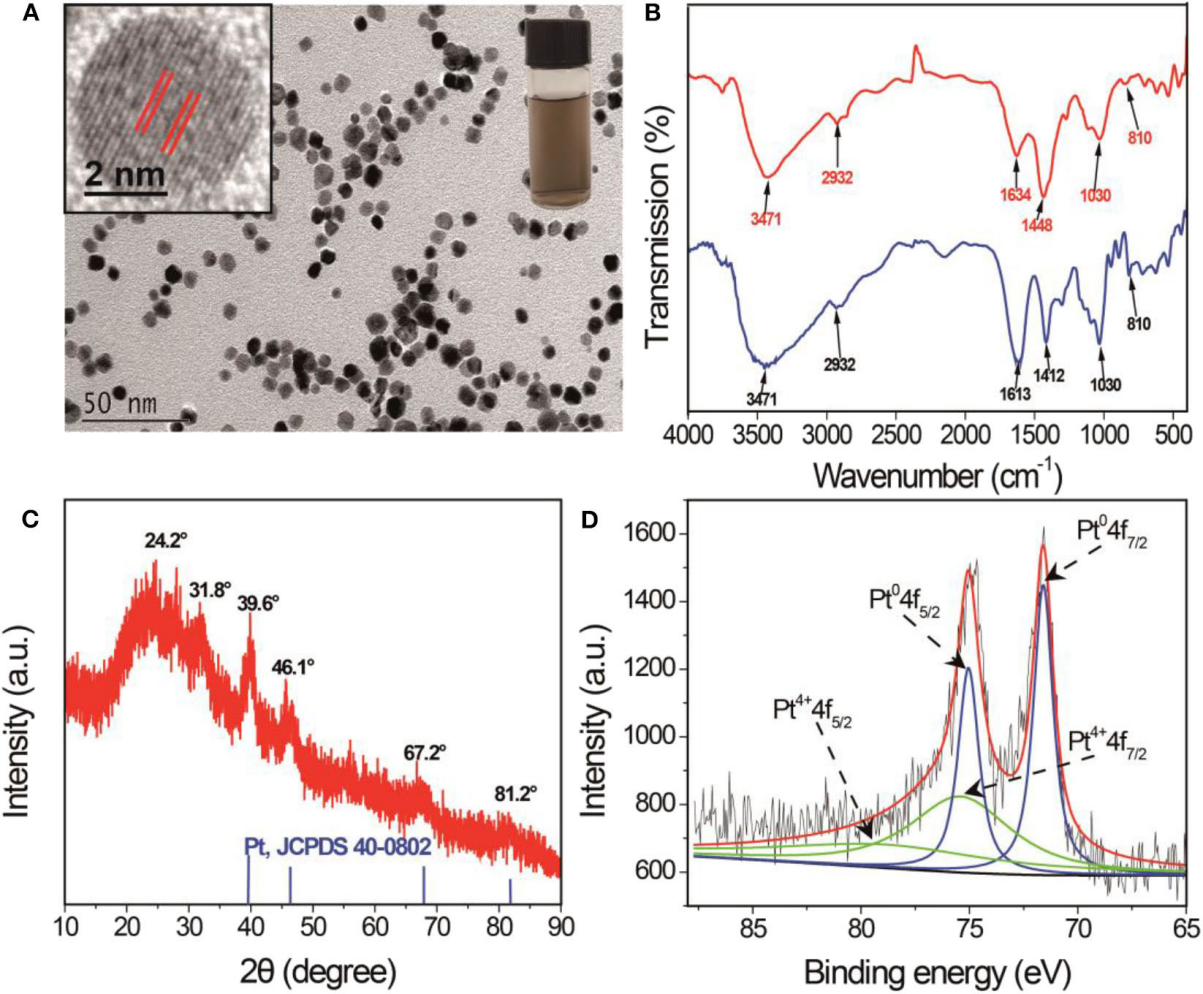
**(A)** TEM image of SA-PtNPs. Inset: The image and high-resolution TEM image of SA-PtNPs. **(B)** Infrared spectra of SA and SA-PtNPs. **(C)** XRD spectra of SA-PtNPs. **(D)** XPS spectra in the Pt 4f region of SA-PtNPs.

SA-PtNPs were prepared by a simple approach with SA as a stabilizing agent and NaBH_4_ as a reductant. The stabilizing and reducing agents have pivotal effects during the synthesis of SA-PtNPs, thus the concentrations of SA and NaBH_4_ had to be determined, respectively (Supplementary Figure 1). As shown in Supplementary Figure 1, the concentrations of SA and NaBH_4_ on the preparation of SA-PtNPs were optimized to be 2 mg/mL and 1.4 mM. The images in Figure 1A display a yellowish-brown product and a state of well-dispersion. Moreover, the high-resolution transmission electron microscope (TEM) image shows that the crystal plane spacing of the SA-PtNPs is 0.19 nm. The size distribution of SA-PtNPs determined from 100 random nanoparticles is shown in Supplementary Figure 2A with an average diameter of 5.9 ± 0.6 nm. The energy dispersive X-ray spectroscopy (EDS) characterization results of SA-PtNPs (Supplementary Figure 2B) indicate that Pt has a peak at the corresponding elemental feature, indicating the presence of Pt in the material. The infrared spectra of SA and SA-PtNPs are shown in Figure 1B. It can be seen that the stretching vibration band of O-H at 3,471 cm^−1^, the stretching vibration band of C-H at 2,932 cm^−1^, and the glycoside skeleton absorption peaks of C-O-C at 1,030 and 810 cm^−1^ are invariable. However, the peaks suggesting -COO- in the molecule at 1,613 and 1,412 cm^−1^ shift to higher wavenumbers of 1,634 cm^−1^ and 1,448 cm^−1^, respectively. These changes probably suggested that the -COO- groups of SA are responsible for interacting with PtNPs.

As shown in Figure 1C, X-ray diffraction (XRD) characterization of SA-PtNPs at 39.6°, 46.1°, 67.2°, and 81.2°can be indexed well to the diffraction from (111), (200), (220), and (311) planes of face-centered cubic (fcc) Pt (JCPDS 04-0802), respectively, proving that Pt exists in the crystalline form. Furthermore, the surface elemental analysis of SA-PtNPs was also performed by X-ray photoelectron spectroscopy (XPS). The characteristic spectrum can be mainly decomposed into two pairs of dual states of Pt 4f_7/2_ and Pt 4f_5/2_. These peaks with the intense distinct peak are concentrated in the binding energies around 71.57 and 75.02 eV, which could be attributed to the existence of metallic platinum (Pt^0^). The other peaks at around 75.47 and 78 eV were contributed to the Pt^4+^ (Cho and Ouyang, 2011; Wu et al., 2014), and the relative contents of Pt^0^ and Pt^4+^ are 42.7 and 57.3% (Figure 1D).

### Oxidase-Like Activity of SA-PtNPs

**Figure 2:**
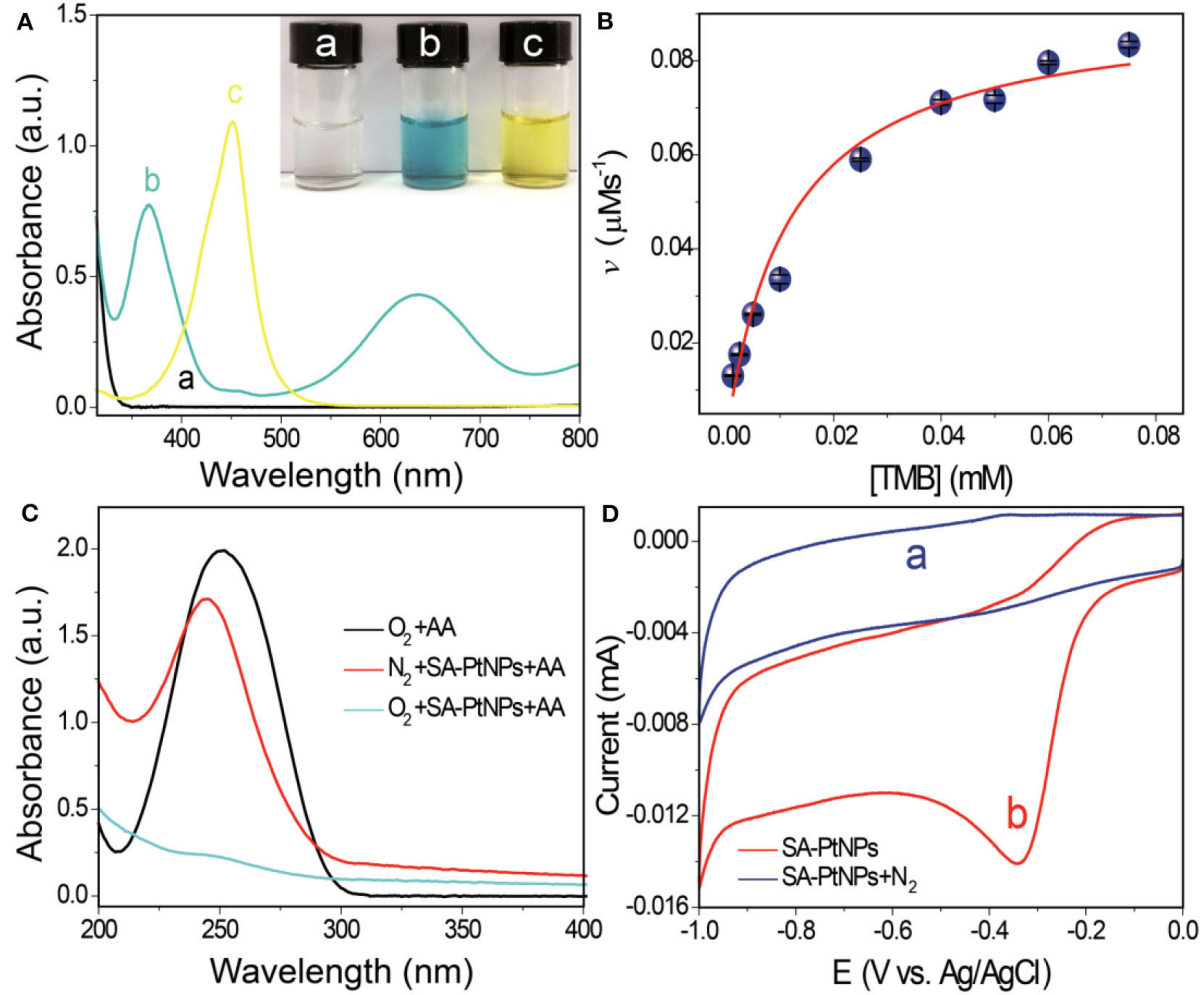
**(A)** UV-vis absorption spectra of (a) TMB, (b) SA-PtNPs + TMB, (c) SA-PtNPs + TMB + stop buffer (H_2_SO_4_). The images show the appearance of the relevant reaction liquid. **(B)** Steady-state dynamic assay of SA-PtNPs. **(C)** Absorption spectra of AA after water bathing for 5 min under different conditions. **(D)** ORR assay, cyclic voltammetry of the ORR on an electrode modified with SA-PtNP in (a) N_2_ and (b) O_2_.

To evaluate the oxidase-like activity of SA-PtNPs, TMB was chosen as the chromogenic substrate in this study. Figure 2A shows that SA-PtNPs can quickly catalyze the oxidation of TMB under O_2_ to generate a blue outcome with peaks absorption at 370 and 652 nm without of H_2_O_2_. Since the absorbance at 450 nm after the addition of H_2_SO_4_ can be stable for a long time with a complete spectrum shift and concomitant hyperchromic effect (Bos et al., 1981; Frey et al., 2000). Therefore, the oxidization reaction in this work was stopped by H_2_SO_4_, resulting in a yellow substance generated with a maximum absorbance at 450 nm. It's worth mentioning that the oxidase-like activity of SA is so low that their effect on SA-PtNPs can be ignored (Supplementary Figure 3).

Some other chromogenic substrates were also tested to demonstrate the versatility of SA-PtNPs, including o-phenylenediamine (OPD), pyrogallol, 2,2′-azino-bis(3-ethylben- zothiazoline-6-sulfonic acid) diammonium salt (ABTS), 4-aminoantipyrine (4-AAP) and N-ethyl-N-(3-sulfopropyl)-3-methylaniline sodium salt (TOPS) (Supplementary Figure 4). Thus, these results verified that SA-PtNPs can act as a new type of oxidase mimic. For further application of the SA-PtNPs-TMB system, pH, temperature, and TMB concentration were optimized to be 4.5, 37°C, and 0.15 mM, respectively (Supplementary Figure 5).

The steady-state dynamics assay of the SA-PtNPs-TMB system is depicted in Figure 2B with the reaction rate (*v*) depending on TMB concentration. With the increase of TMB concentration, reaction rate increased quickly at first and then changed slowly. To further study the kinetics, the Michaelis Constant (*K*_*m*_) value of SA-PtNPs was counted to be 11.6 μM fitting by Michaelis-Menten equation. The *K*_*m*_ is an important parameter used to evaluate the affinity of nanozyme to substrate and to discuss the nanozyme-substrate interaction. Table 1 also shows the comparison of the *K*_*m*_ value of oxidase mimics and points out that SA-PtNPs have a remarkably high affinity for TMB. As shown in Supplementary Figure 6, the specific activity value of SA-PtNP has been evaluated as 2,711 U/g according to the fitted straight line (Supplementary Figure 6B) (Jiang et al., 2018).

**Table 1:** Kinetic parameters for nanzoymes as oxidase mimics.

**Catalyst**	**Substrate**	***K_***m***_***	***v*_**max**_**	**References**
		**(mM)**	**(×10^**−8**^Ms^**−1**^)**	
CeO_2_ NPs	TMB	0.8~3.8	30~70	Asati et al., 2009
MnO_2_ NPs		0.04	578	Liu et al., 2012
Ni/Pd hollow NPs		0.11	1.52	Wang et al., 2016
Lysozyme-PtNPs		0.63	270	Yu et al., 2014
Chitosan-PtNPs		0.018	15.7	Deng et al., 2017
Heparin-PtNPs		0.010	4.962	He et al., 2019b
Heparin-OsNPs		0.8	8.45	He et al., 2019c
Citrate-PtNPs		0.1206	6.51	Wu et al., 2014
SA-PtNPs		0.012	9.145	This work

To determine the mechanism, possible superoxide anions (${\text{O}}_{2}^{•-}$) were also studied. First of all, SA-PtNPs-catalyzed TMB oxidation was conducted in air with dissolved oxygen and bubbled with highly pure nitrogen gas, respectively. The reaction rate of SA-PtNPs-catalyzed TMB oxidation was found to decrease rapidly under nitrogen atmosphere, which indicates that dissolved O_2_ is involved in the process (Supplementary Figure 7). Since ascorbic acid (AA) could eliminate ${\text{O}}_{2}^{•-}$ (Cai et al., 2018), we performed AA oxidations. As Figure 2C shows that AA has an absorption peak at ~250 nm in the wavelength range. A slight variation at 250 nm and oxidation of AA occurred while SA-PtNPs or O_2_ was removed. However, when SA-PtNPs and O_2_ co-exist, AA was consumed. In this phenomenon, the presence of ${\text{O}}_{2}^{•-}$ can be ruled out in the SA-PtNPs-TMB system. Furthermore, the oxygen reduction reaction (ORR) assay was carried out to further verify the mechanism. Relevant experiments were carried out with using carbon electrode as electrochemical working electrode which was modified with SA-PtNPs. No peak appear after the mixed solution is filled with nitrogen (Figure 2D). It produced a cathode reduction peak at ~-0.34 V in an environment with oxygen. On the basis of these results, the oxidase-like activity of SA-PtNPs originates mainly from the formation of ${\text{O}}_{2}^{•-}$ during the reaction.

### The Contribution of SA in SA-PtNPs

**Figure 3:**
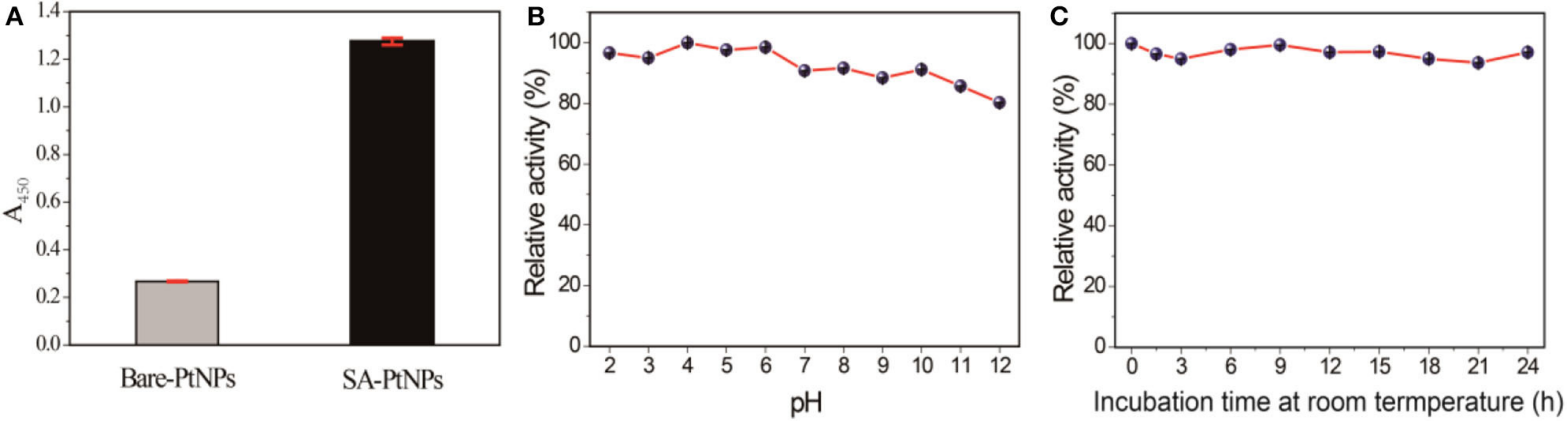
**(A)** Comparison of oxidase activity between SA-PtNPs and bare PtNPs. Relative activity of SA-PtNPs under different **(B)** pH values and **(C)** incubation time. (The maximum value was defined as 100% relative activity).

Several experiments have been carried out to investigate the contribution of SA in SA-PtNPs. First of all, we found that bare PtNPs show distinct aggregation in solution, while the SA-PtNPs exhibited excellent colloidal stability when SA was introduced into the preparation process (Supplementary Figure 8). Compared with bare platinum without any protective agent, SA-PtNPs obtain a much higher oxidase-like activity that implies the improvement effect with the introduction of SA (Figure 3A). As is generally known, the stability of PtNPs against aggregation under different harsh conditions is crucial to the extension of the practical application. To this end, SA-PtNPs were exposed to several pH values (2–12) for 2 h to explore the effect of pH on the oxidase activity. As shown in Figure 3B, the oxidase-like activity of SA-PtNPs fluctuated slightly with the change of environment pH, implying excellent pH stability of SA-PtNPs. Furthermore, we found that SA-PtNPs possessed perfect oxidase-like activity even if they have been stored at room temperature for a long time (Figure 3C). The above results confirm that the contribution of SA in PtNPs preparation increases the oxidase-like activity and stability of PtNPs.

### Antioxidant Capacity Evaluation and Colorimetric Assay for OPC

The phenolic hydroxyl groups in the OPC structure make it a good hydrogen donor and therefore, it has a strong antioxidant capacity (Shao et al., 2018). Owing to the scavenging ability of OPC to ${\text{O}}_{2}^{•-}$ and the formation of ${\text{O}}_{2}^{•-}$ in the SA-PtNPs-TMB system, it is speculated that OPC could inhibit the catalytic process. Thus, a colorimetric assay for OPC was established (Scheme 1).

**Figure 4:**
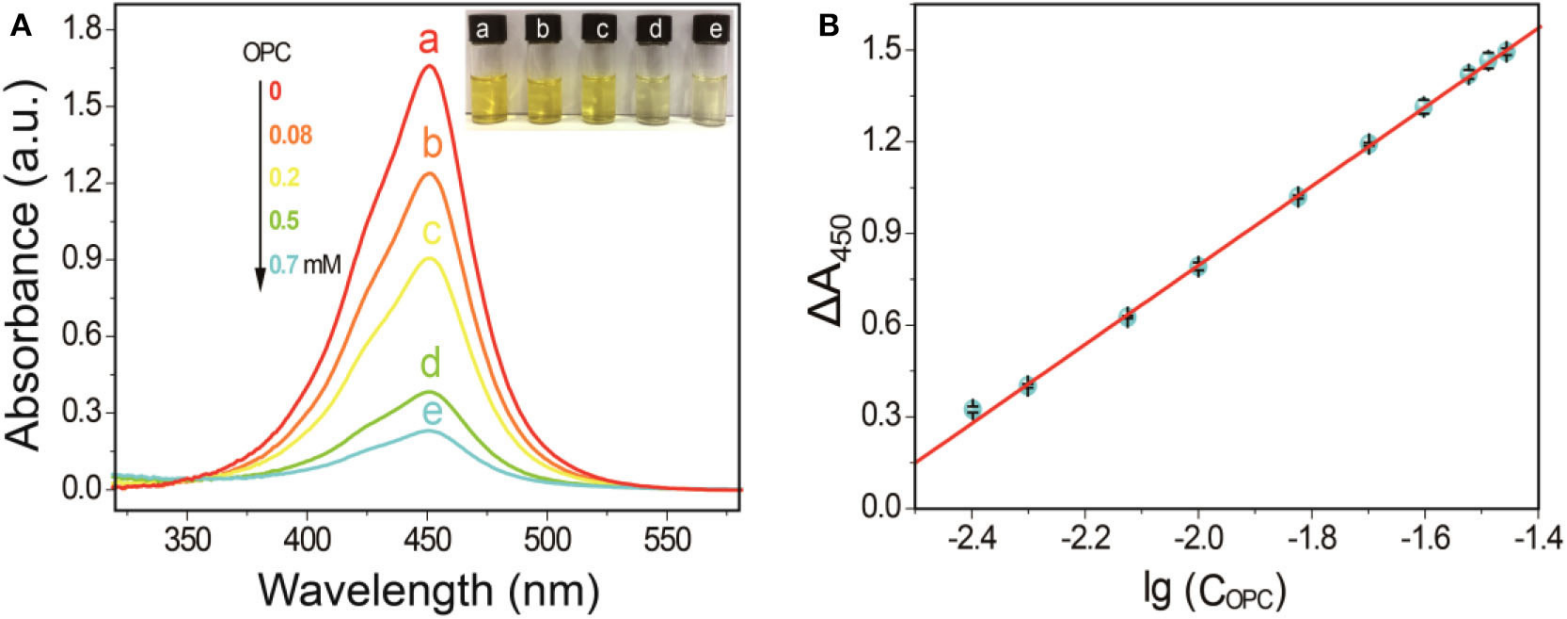
**(A)** Inhibitory effect of OPC at different concentrations on simulated enzyme activity. **(B)** The linearity between ΔA_450nm_ and the logarithmic value of OPC concentration.

Experimental results in Figure 4A indicated the A_450nm_ of the SA-PtNPs-TMB system gradually decreased as the concentration of OPC increases. When the concentration of OPC in the reaction system reached 1 mM, the oxidation of TMB by SA-PtNPs was drastically inhibited. These phenomena fully illustrate that OPC could distinctly inhibit the color response of the SA-PtNPs-TMB system, namely inhibiting the oxidase-like activity of SA-PtNPs. Linearity was obtained by plotting the value of ΔA_450nm_ and the logarithmic value of OPC concentration (Figure 4B). (ΔA_450nm_=A_0_-A_t_, where A_0_ and A_t_ are the absorbance of the reaction product at 450 nm in the inexistence and existence of OPC, respectively.) The range of linearity was from 4 to 32.5 μM, the relative standard deviation (RSD) is 0.6% for measuring 10 μM, and limit of detection (LOD) for OPC was 2.0 μM (ΔA_450nm_=1.1791 lgC_OPC_ + 3.1941, r = 0.999). As shown in Supplementary Table 1, most reported methods use high-performance liquid chromatography or mass spectrometry technology to analyze PC or OPC and are time-consuming, expensive, and technically demanding. What's more, the proposed method showed a comparable sensitivity and LOD to those of the reported methods.

Based on this linear equation, the antioxidant capacity of OPC was also assessed. Before measurements, OPC extracted from grape seeds was centrifuged to remove any solid residue (Soxhlet extraction method, Supplementary Material). AA is a well-recognized substance with antioxidant capacity. In this study, the inhibitory effect of 1 mM AA on the SA-PtNPs-TMB system is defined as 1 U. Therefore, the antioxidant capacity of pure OPC was calculated to be 2.85 U/mg (Supplementary Material). More significantly, as shown in Table 2, the recoveries of the standard addition experiment in grape seeds ranged from 97.0 to 98.6% with RSD values of 0.5–3.4%. These results provide a new insight for antioxidant capacity and indicate the accuracy of this assay for OPC analysis.

**Table 2:** Recovery of standard addition of OPC in grape seeds.

**Sample**	**Measured before addition (μM)**	**Added (μM)**	**Found (μM)**	**Recovery (%)**	**RSD (%, *n* = 3)**
Grape seeds	11.1	10	9.9	98.6	2.4
		15	14.8	98.4	0.5
		20	19.4	97.0	3.4

## Conclusions

A facile one-step synthesis method was successfully introduced to prepare SA-PtNPs (5.9 ± 0.6 nm) using SA as a surface modification agent and NaBH_4_ as the reducing agent. SA-PtNPs can catalyze the oxidation of the chromogenic substrate TMB by dissolved oxygen and has oxidation simulation enzyme activity. These phenomena demonstrate that SA-PtNPs can perform as a highly efficient and robust oxidase mimic for the catalyzing substract-O_2_ reaction. The steady-state kinetic assay has shown that SA-PtNPs have a stronger affinity to TMB than those of most nanozymes. Additionally, the specific activity of SA-PtNPs has been calculated to be 2,711 U/g. The wide pH value and incubation time of the reaction environment have little effect on the oxidase-like activity of SA-PtNPs. Therefore, SA not only increases the dispersibility and stability of PtNPs but also improves the catalytic activity of PtNPs as oxidase mimics. More significantly, results have shown that OPC can effectively prevent oxidation of TMB and have an inhibitory effect on the color development system. The colorimetric assay for OPC in grape seeds established by this phenomenon has good linearity, reproducibility, low limit of detection, and is easy to perform. The antioxidant capacity of pure OPC obtained by the Soxhlet extraction method was 2.85 U/mg. This assay addresses the shortage of OPC methods and extends the potential application of platinum nanozymes.

## Data Availability Statement

All datasets generated for this study are included in the article/Supplementary Material.

## Author Contributions

Conceptualization: WC, H-HD, and G-LH. Methodology: G-LH, H-PP, and ZL. Investigation: S-BH, LY, and X-LL. Writing—original draft preparation: S-BH. Writing—review and editing: WC and H-HD. Funding acquisition: WC. All authors have read and agreed to the published version of the manuscript. All authors contributed to the article and approved the submitted version.

## Conflict of Interest

The authors declare that the research was conducted in the absence of any commercial or financial relationships that could be construed as a potential conflict of interest.
