# Supplementary material for: Sodium Alginate Modified Platinum Nanozymes With Highly Efficient and Robust Oxidase-Like Activity for Antioxidant Capacity and Analysis of Proanthocyanidins
Source: Front Chem. 2020 Aug 5;8:654. doi: 10.3389/fchem.2020.00654 (PMC7419988; doi:10.3389/fchem.2020.00654)
Supplement: Supplementary file 1 [file Table_1.DOCX]

**SUPPLEMENTARY MATERIAL**

**Sodium Alginate Modified Platinum Nanozymes with Highly Efficient and Robust Oxidase-Like Activity for Antioxidant Capacity and Analysis of Proanthocyanidins**

Shao-Bin He,^a^ Liu Yang,^a^ Xiu-Ling Lin,^a^ Hua-Ping Peng,^a^ Zhen Lin,^a^ Hao-Hua Deng,^a^* Wei Chen,^a^* Guo-Lin Hong^b^*

^a^ Fujian Key Laboratory of Drug Target Discovery and Structural and Functional Research, School of Pharmacy, Fujian Medical University, Fuzhou 350004, China

^b^ Department of Laboratory Medicine, The First Affiliated Hospital of Xiamen University, Xiamen 361005, China

* Corresponding author. Tel./fax: +86 591 22862016.

E-mail address: chenandhu@163.com (W. Chen), DHH8908@163.com (H-H Deng) , 18860089899@139.com (G-L Hong).

**EXPERIMENTAL PART**

**Materials and Reagents**

SA was bought from Jingchun Reagent Co., Ltd. (Shanghai, China). NaBH_4_, H_2_PtCl_6_, TMB, o-phenylenediamine (OPD), pyrogallol, 2,2’-azino-bis(3-ethylben- zothiazoline-6-sulfonic acid) diammonium salt (ABTS), 4-aminoantipyrine (4-AAP) and N-ethyl-N-(3-sulfopropyl)-3-methylaniline sodium salt (TOPS) were purchased from Aladdin Reagent Co. Ltd (Shanghai, China). [Ascorbic](D:/Youdao/Dict/8.5.1.0/resultui/html/index.html#/javascript:;) [acid](D:/Youdao/Dict/8.5.1.0/resultui/html/index.html#/javascript:;), Na_2_HPO_4_⋅12H_2_O, NaH_2_PO_4_⋅2H_2_O, H_3_PO_4_, and H_2_SO_4_ were obtained from Sinopharm Chemical Reagent Co., Ltd. (Shanghai, China). The OPC was bought from Yuanye Biotechnology Co., Ltd. (Shanghai, China).

**Apparatus and Characterization**

The ultraviolet-visible (UV-vis) absorption spectra were recorded using a UV-2450 UV-vis spectrophotometer (Shimadzu Corporation, Kyoto, Japan). Transmission electron microscope (TEM) images were collected with a JEM-2100 TEM (JEOL, Japan). X-ray photoelectron spectroscopy (XPS) was performed on an ESCALAB 250XI electron spectrometer (Thermo Fisher Scientific Inc., USA). Infrared (IR) spectra were measured at wavenumbers of 400–4000 cm^-1^ using a Nicolet Avatar 360 Fourier transform (FT)-IR spectrophotometer (Thermo Fisher Scientific Inc., USA). X-ray diffraction (XRD) patterns were recorded with a Bruker D8 Advance diffractometer (Germany). Electrochemical measurements were conducted with an electrochemical workstation (CHI660C, Shanghai Chenhua Device Company, China).

**Oxidase-Like Activity**

50 μL of TMB (3 mM) and 30 μL of SA-PtNPs (7.803 mg/L) were added into 920 μL of PB buffer (pH=4.5, 50 mM). Then, the mixture was incubated in 37 °C bath for 5 min. Subsequently, 200 μL of H_2_SO_4_ (2 M) was introduced to the reaction as stop buffer. Finally, the solution was transferred to a quartz cell for measurement at 450 nm.

20 μL of OPD (0.1 M) and 30 μL of SA-PtNPs (7.803 mg/L) were added into 950 μL of PB buffer. Then, the mixture was incubated in 37 °C bath for 5 min. Subsequently, the solution was transferred to a quartz cell for measurement.

20 μL of pyrogallol (0.1 M) and 30 μL of SA-PtNPs (7.803 mg/L) were added into 950 μL of PB buffer. Then, the mixture was incubated in 37 °C bath for 5 min. Subsequently, the solution was transferred to a quartz cell for measurement.

20 μL of ABTS (0.1 M) and 30 μL of SA-PtNPs (7.803 mg/L) were added into 950 μL of PB buffer. Then, the mixture was incubated in 37 °C bath for 5 min. Subsequently, the solution was transferred to a quartz cell for measurement.

10 μL of 4-AAP (0.1 M), 10 μL of TOPS (20 mM) and 30 μL of SA-PtNPs (7.803 mg/L) were added into 950 μL of PB buffer. Then, the mixture was incubated in 37 °C bath for 5 min. Subsequently, the solution was transferred to a quartz cell for measurement.

**Oxygen Reduction Reaction**

Pt wire was used as the counter electrode, and Ag/AgCl (KCl saturation) was the reference electrode. An aqueous solution of 50 μL of SA-PtNPs was dropped on a carbon screen-printed electrode. On this basis, the SA-PtNPs-modified electrode was air-dried at room temperature for 1 h. Subsequently, 1 μL of 0.5% Nafion solution was deposited on each electrode. Prior to each electrochemical test, the electrolyte solution (0.1 M KOH) was bubbled with N_2_ or O_2_ for 20 min. Cyclic voltammetry was performed at a scan rate of 50 mV s^-1^ over a potential range of −1.0 to 0.0 V.

**Extraction and Purification of OPC**

The grape seeds were first ground and pulverized, and then extracted with a Soxhlet extractor (using petroleum ether as a solvent, refluxing 8 times per hour, and extracting for 12 h, until the petroleum ether was evaporated and used). Then 60% ethanol was used as a solvent (the ratio of material to liquid was 1:7) and leached three times for 30 minutes in a 48.6°C water bath rotary shaker. After cooling, centrifuge at 4000 r/m in for 20 min, and combine the supernatant to obtain a crude extract. The crude extract was concentrated in vacuo using a rotary evaporator at 40°C under a vacuum of 0.095 MP. The concentrate was applied to a well-treated macroporous resin chromatography column and eluted with water and 20%, 40%, 60%, 80% ethanol, respectively. Thereafter, the eluate was separately concentrated in a vacuum and dried. The dried product was dissolved in a small amount of 75% acetone, mixed with a small amount of polyamide powder, and then dry-coated, and eluted with an acetone:water = 3:1 eluent at a rate of 2 mL/min. A partial collector was used to collect one tube every 2 min. Thereafter, the fraction of the eluate containing OPC was combined, concentrated under vacuum and dried to obtain the OPC extract.

**Antioxidant Capacity Evaluation**

50 μL of samples (7.625 g/L), 50 μL of TMB (3 mM) and 30 μL of SA-PtNPs (15.61 mg/L) were added into 870 μL of phosphate buffer (pH=4.5, 50 mM). The mixture was then incubated for 5 min at 37 °C. Subsequently, 200 μL of H_2_SO_4_ (2 M) was introduced to the reaction as a stop buffer. Finally, the solution was transferred to a quartz cell for measurement at 450 nm. The concentration of OPC (C_1_) was calculated to be 0.0035 mM by the linear equation (ΔA_450_=1.326lgC_OPC_+3.4538).

50 μL of ascorbic acid (20 mM), 50 μL of TMB (3 mM) and 30 μL of SA-PtNPs (15.61 mg/L) were added into 870 μL of phosphate buffer (pH=4.5, 50 mM). The mixture was then incubated for 5 min at 37 °C. Subsequently, 200 μL of H_2_SO_4_ (2 M) was introduced to the reaction as a stop buffer. Finally, the solution was transferred to a quartz cell for measurement at 450 nm. A concentration (C_2_) was calculated to be 0.0032 mM by the linear equation (ΔA_450_=1.326lgC_OPC_+3.4538).

The inhibitory effect of 1 mM AA on the SA-PtNPs-TMB system is defined as 1 U.

The antioxidant capacity of OPC = (C_1_ / C_2_ ) / m= (0.0035/0.0032 U) / 7.625=2.85 U/mg.

**The calculation of LOD**

The experiment of blank group was repeated to determine the fluctuation range of blank value. Then, ΔA_0_ is calculated. (ΔA_0_=A_0_’-A_0_, where A_0_'and A_0_ are the absorbance of the reaction product at 450 nm when OPC is absent, A_0_ was measured when establishing the working curve). Further, calculate the standard deviation of ΔA_0_ (σ = 0.00251). Finally, 3 times of the standard deviation (i.e. 3σ = 0.00753) is substituted into the linear equation (ΔA_450_ =1.1791 lgC_OPC_ + 3.1941). Then, the LOD was calculated to be 0.002 mM by the linear equation (LOD = 10^[(3σ -3.1941)/1.1791]=0.002 mM ).


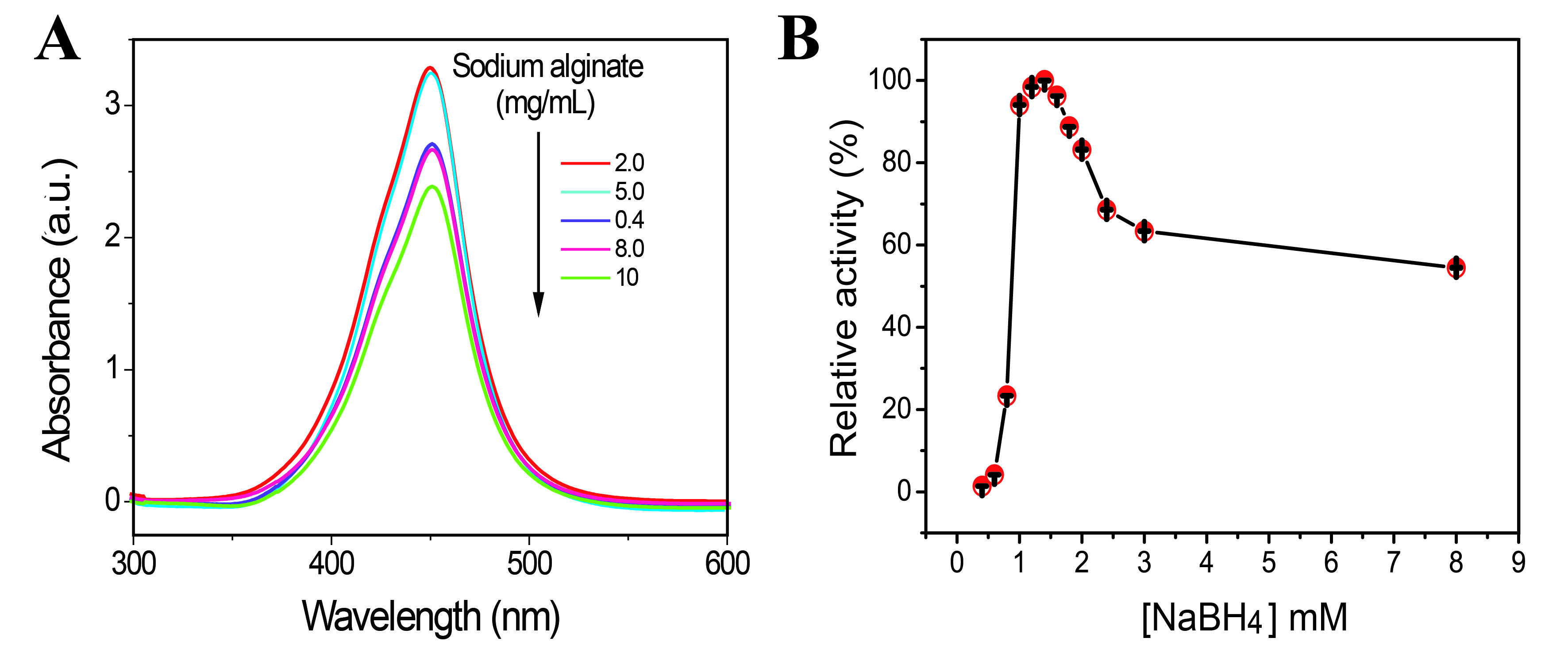


**SUPPLEMENTARY FIGURE S1. |** Effects of SA **(A)** and NaBH_4_ **(B)** on the catalytic activity of SA-PtNPs. (50 μL of 3 mM TMB and 17.5 μL of SA-PtNPs (78.03 mg/L) were added into 932.5 μL of PB buffer (pH=4.5, 50 mM). This solution was incubated in a 37 °C bath for 5 min. Subsequently, 200 μL of 2M H_2_SO_4_ was introduced to terminate the reaction. Finally, the mixture was transferred into a quartz cell to measure the absorbance at 450 nm.)


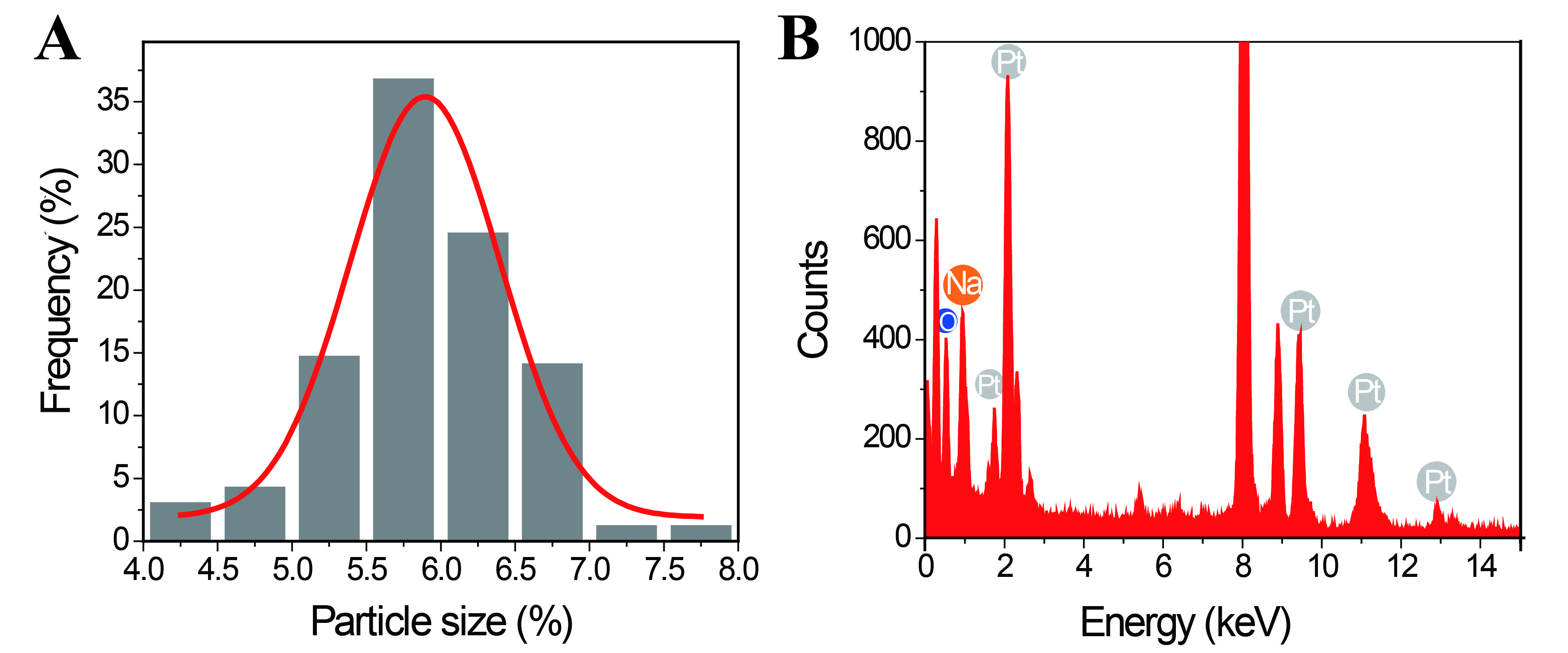


**SUPPLEMENTARY FIGURE S2 | (A)** The size distribution of SA-PtNPs determined from size distribution analysis of 100 random nanoparticles by Gaussian fitting. **(B)** EDS spectrum of SA-PtNPs.


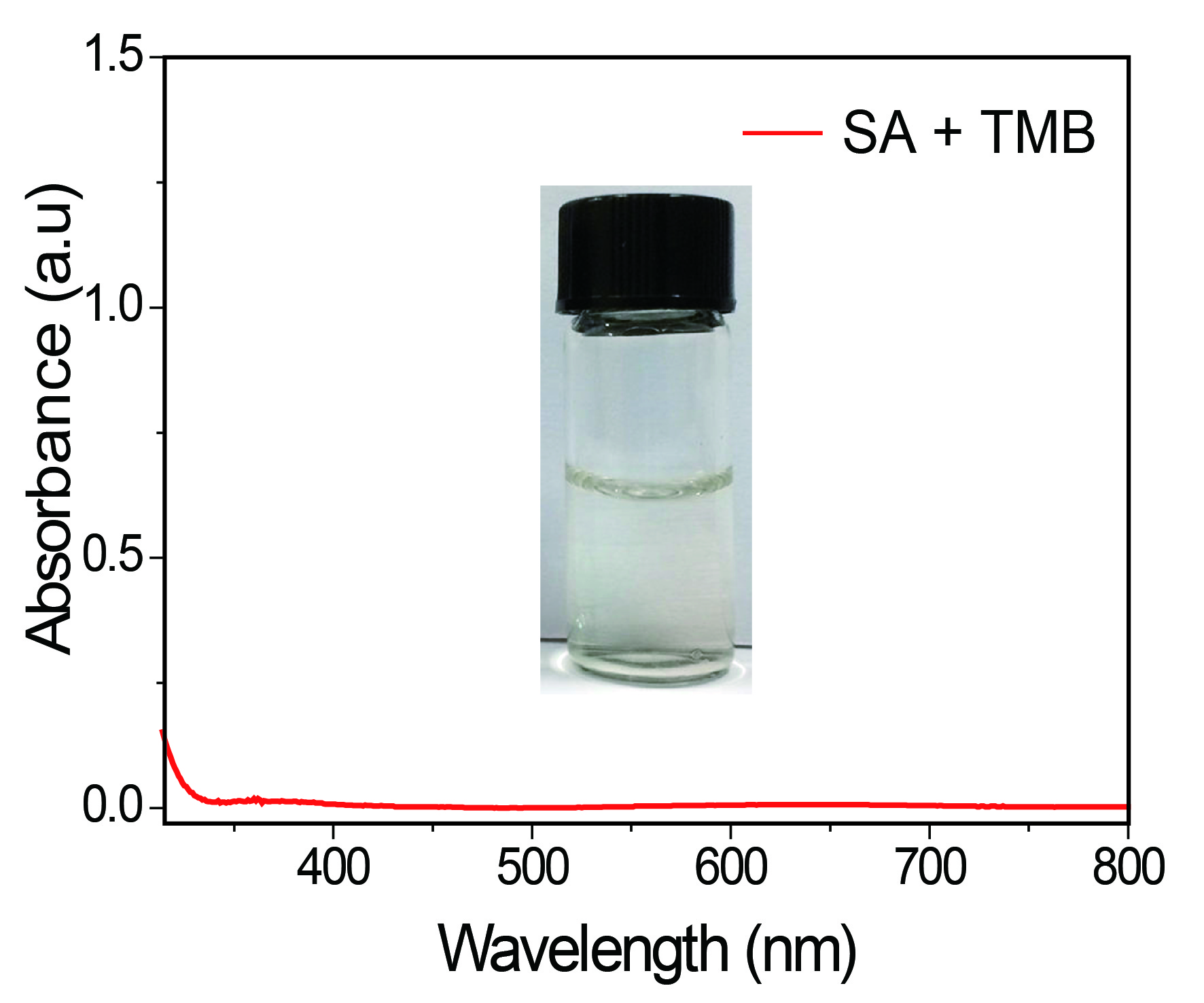


**SUPPLEMENTARY FIGURE S3 |** UV/vis spectra of TMB+SA.


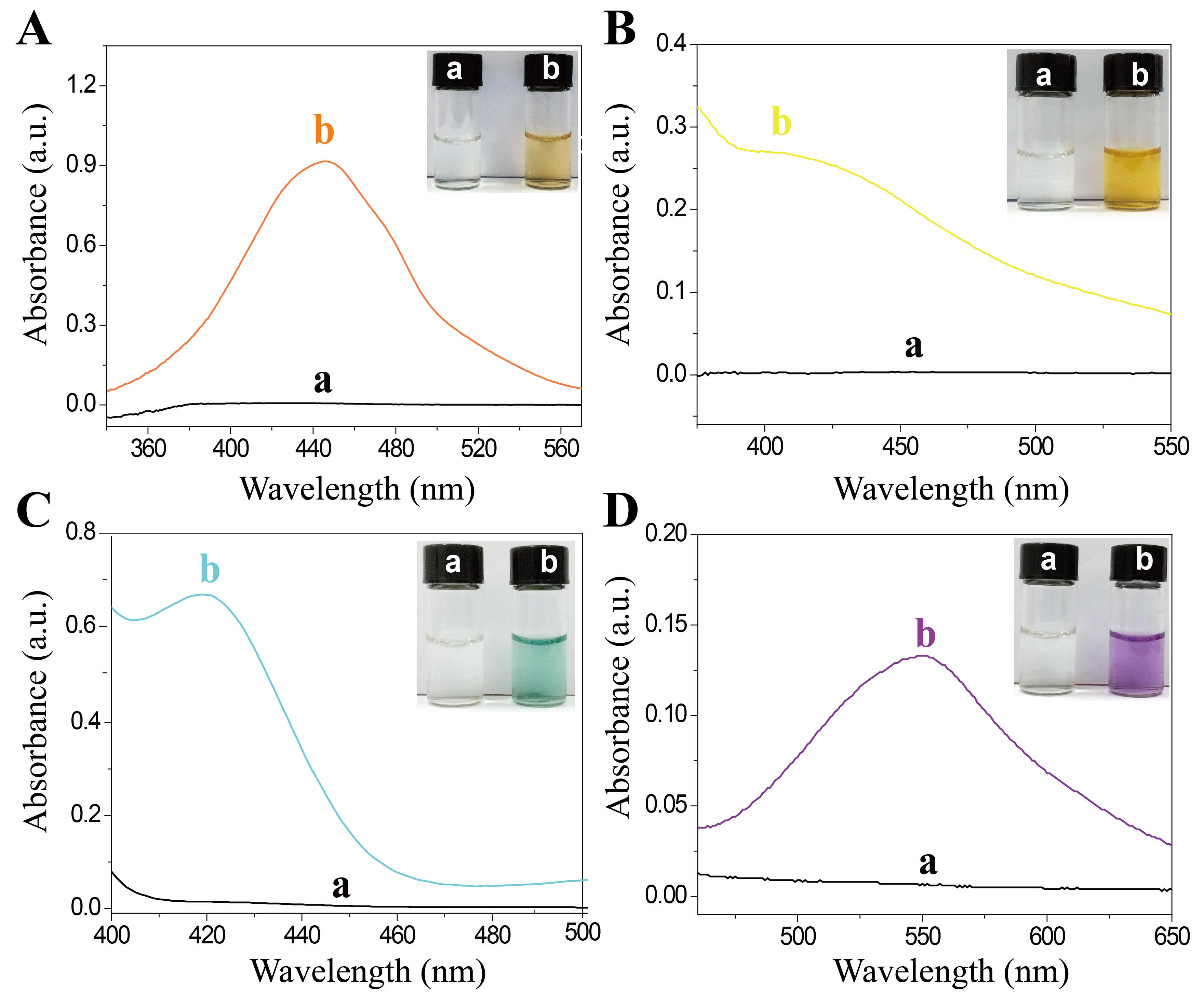


**SUPPLEMENTARY FIGURE S4 |** UV-vis spectra of **(A)** OPD, **(B)** pyrogallol, **(C)** ABTS, and **(D)** 4AAP/TOPS catalyzed by SA-PtNPs: **(a)** substrate only, **(b)** substrate + SA-PtNPs. Inset: the corresponding images.


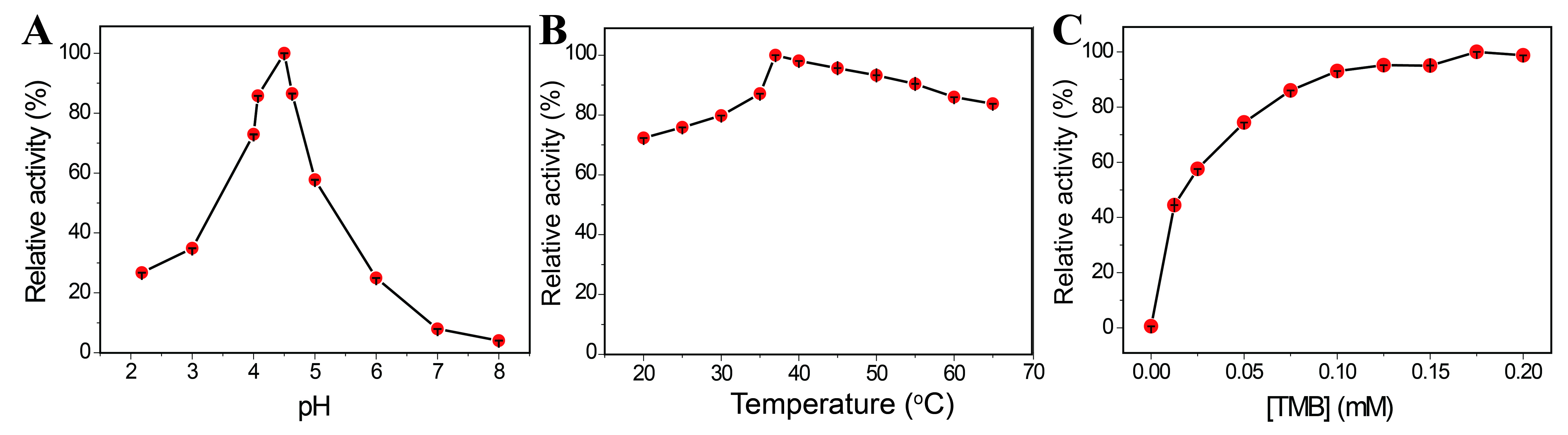


**SUPPLEMENTARY FIGURE S5 |** The optimization of SA-PtNPs-TMB reaction including **(A)** pH, **(B)** temperature optimization, **(C)** TMB concentration. (The highest point was defined as 100% relative activity)


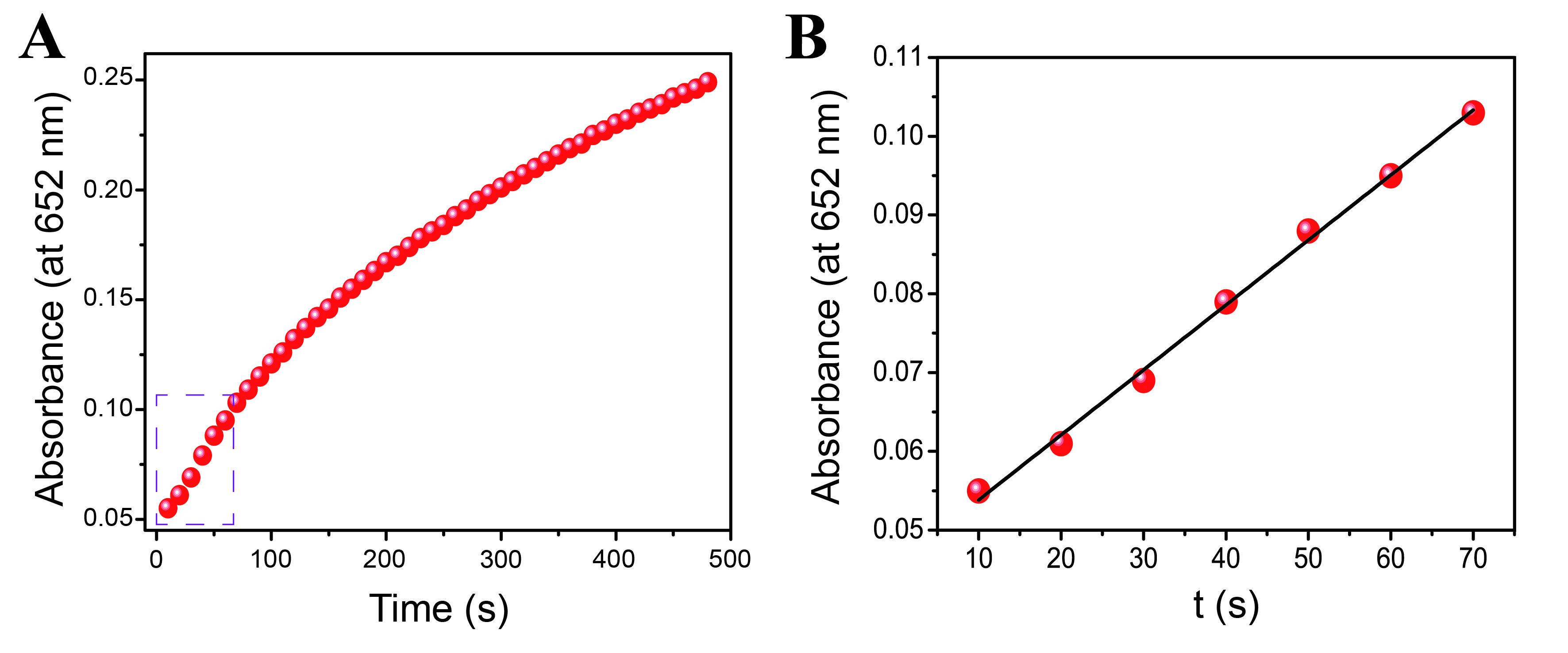


**SUPPLEMENTARY FIGURE S6 | (A)** Standardization of oxidase-like activity of SA-PtNPs; **(B)** Magnified initial linear portion of the reaction time curves. A time length of 70 s was chosen for the initial rate period because the coefficients were close to 1 after a linear regression analysis. (One unit is defined as the amount of nanozyme that catalyzes 1 μmol of product at 25 ^o^C per minute. The specific activity is defined as activity units per gram of nanozyme.)

**Supplementary Figure S6** is the curve of the TMB color reaction catalyzed by SA-PtNPs. First of all, SA-PtNPs (15.61 mg/L) responded with TMB (0.15 mM) at 25 ^o^C. Then, the absorbance of the mixture was measured every 10 s. Reaction-time curves were obtained by plotting the absorbance at 652 nm against the reaction time(see **Supplementary Figure S6A**). The reaction-time curve of the first 70 s in the reaction process was fitted (**Supplementary Figure S6B**). Then, calculate the nanozyme activity (units) using the following equation (Jiang et al., 2018):

*b*_nanozyme_ = $\frac{V}{\varepsilon\times l}\times\frac{\text{ΔA}}{\text{Δt}}$

Here, *b*_nanozyme_ represents the catalytic activity unit of nanozyme; V is the total volume of solution (μL); ε is the molar absorption coefficient of the colorimetric substrate TMB; l stands for optical path (cm); ΔA/Δt(a.u./min) represents the rate of change of absorbance with time. (V = 1000 μL, ε_TMB_ = 39000 M^-1^cm^-1^, l = 1 cm, ΔA/Δt = 8.25 × 10^-4^ a.u./s）

So, *b*_nanozyme =_ 1000/39000 × (8.25× 10^-4^/60) = 1.269 × 10^-3^ U.

The specific activity (SA) is defined as activity units per gram of nanozyme here. SA_SA-PtNPs_=*b*_nanozyme_/m_nanozyme_ = 1.269 × 10^-3^ U / (15.61 mg/L× 30 μL) = 2711 U/g.


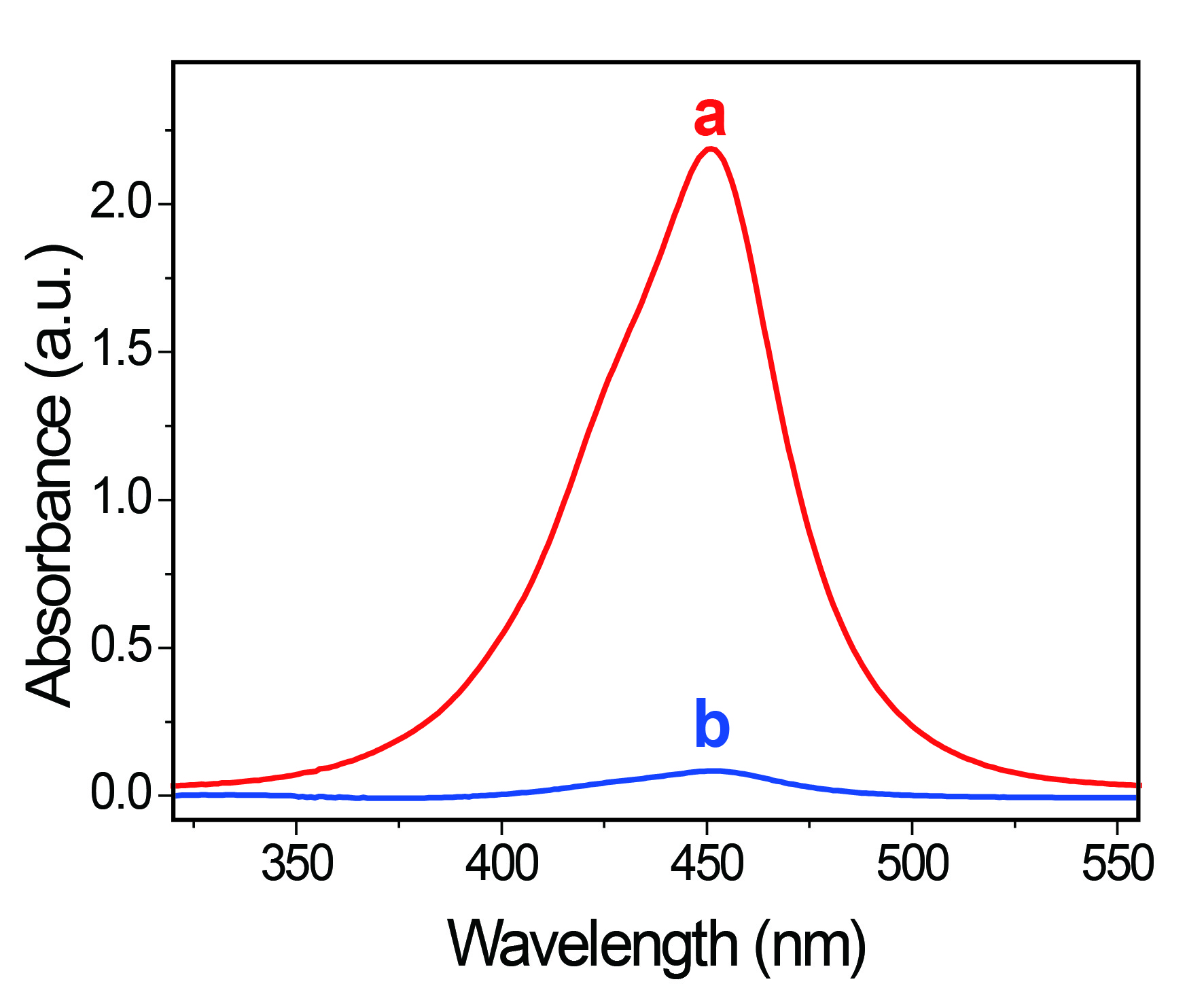


**SUPPLEMENTARY FIGURE S7 |** SA-PtNPs-catalyzed TMB oxidation was conducted in air with (a) dissolved oxygen and (b) after the reaction mixture was bubbled with highly pure nitrogen gas for 10 min.

**A B**


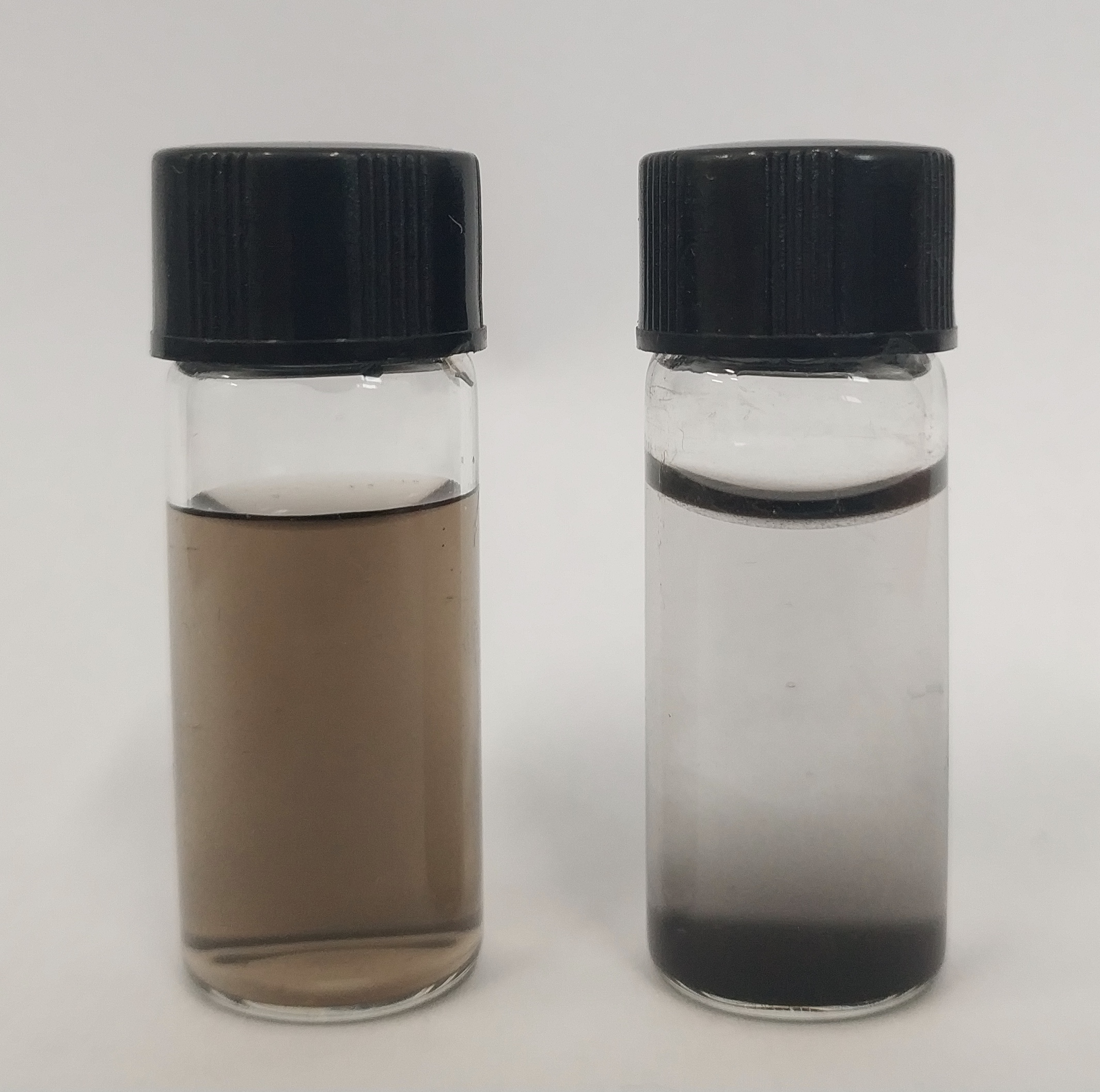


**SUPPLEMENTARY FIGURE S8 |** Photographs of (A) SA-PtNPs and (B) bare PtNPs. (The synthesis steps of bare PtNPs are as follows: 2 mL H_2_PtCl_6_ (10 mM) was mixed with of 1% (v/v) acetic acid solution. The mixture was vortex at ambient temperature for 30 min. 1 mL of [newly-prepared](http://dict.cnki.net/javascript:showjdsw('showjd_2','j_2')) NaBH_4_ solution (70 mM) was added to the mixture and was completed within 5 min. The bare PtNPs were obtained by stirring in the dark for 90 min. )

**SUPPLEMENTARY TABLE S1 |** Comparison of reported methods for probing proanthocyanidins.

| **Methods** | **Linear range (mg/L)** | **LOD (mg/L)** | **Ref.** |
| --- | --- | --- | --- |
| thiolysis HPLC | 50-100 | 17 | Gao et al., 2018 |
| HPLC-ESI-QTOF-MS | 0.39-6.25 | 0.096 | Cádiz-Gurrea et al., 2017 |
| HPLC-DAD-MS/MS | - | 0.00476 | García-Estévez et al., 2017 |
| HPLC-DAD-MS | - | 0.17 | Gris et al., 2011 |
| Spectrophotometry  (MW=468.42) | 1.87-15.22 | 0.937 | This work |
|  | 4-32.5 μM | 2.0 μM |  |

**REFERENCE**

Cádiz-Gurrea, M.D.L.L., Borrás-Linares, I., Lozano-Sánchez, J., Joven, J., Fernández-Arroyo, S., and Segura-Carretero, A. (2017) Cocoa and grape seed byproducts as a source of antioxidant and anti-Inflammatory proanthocyanidins. *Int. J. Mol. Sci.* 18, 376-389. doi: 10.3390/ijms18020376

Gao, C., Cunningham, D.G., Liu, H., Khoo C., and Gu L. (2018) Development of a thiolysis HPLC method for the analysis of procyanidins in cranberry products. *J. Agr. Food Chem.* 66, 2159-2167. doi: 10.1021/acs.jafc.7b04625

García-Estévez, I., Alcalde-Eon, C., and Escribano-Bailon, M.T., (2017) Flavanol quantification of grapes via multiple reaction monitoring mass spectrometry. application to differentiation among clones of vitis vinifera l. cv. rufete grapes. *J. Agr. Food Chem.* 65, 6359-6368. doi: 10.1021/acs.jafc.6b05278

Gris, E.F., Mattivi, F., Ferreira, E.A., Vrhovsek, U., Pedrosa, R.C., and Bordignon-Luiz M.T. (2017) Proanthocyanidin profile and antioxidant capacity of Brazilian Vitis vinifera red wines. [*Food Chem.*](http://xueshu.baidu.com/usercenter/data/journal?cmd=jump&tn=SE_baiduxueshu_c1gjeupa&ie=utf-8&sc_f_para=sc_hilight=publish&sort=sc_cited&wd=journaluri:(482e10c41cb246dc)%20Food%20Chemistry) 126 p.213-220. doi: 10.1016/j.foodchem.2010.10.102

Jiang, B., Duan, D., Gao, L., Zhou, M., Fan, K., Tang, Y., et al. (2018) Standardized assays for determining the catalytic activity and kinetics of peroxidase-like nanozymes. *Nat. Protoc.* 13, 1506-1520. doi: 10.1038/s41596-018-0001-1
